# Investigating the capability of the structure-from-motion photogrammetry on monitoring the ice surface sublimation

**DOI:** 10.1016/j.isci.2026.114775

**Published:** 2026-01-24

**Authors:** Junfeng Liu, Shaoxiu Ma, Rensheng Chen, Yongyuan Li, Xueliang Wang, Chuntan Han

**Affiliations:** 1 State Key Laboratory of Ecological Safety and Sustainable Development in Arid Lands, Northwest Institute of Eco-Environment and Resources, Chinese Academy of Sciences, Lanzhou, China; 2 Qilian Alpine Ecology and Hydrology Research Station, Northwest Institute of Eco-Environment and Resources, Chinese Academy of Sciences, Lanzhou, China; 3 Qilian Forestry and Grassland Administration/Qilian Management Branch of Qilian Mountain National Park (Qinghai), Xining, Qilian County 810400, China; 4 Gansu Hydrological Station, Lanzhou, China

**Keywords:** Earth sciences, Geography, Physical geography, Remote sensing

## Abstract

Sublimation is an important process of moisture and energy exchange on the surface of ice and snow, but it is difficult to obtain observational data in winter using the traditional manual weighing method and eddy covariance, which hinders the research of the sublimation process and its mechanism on the ice surface. This study investigates the accuracy of the time-lapse Structure-from-Motion photogrammetry method (O-T-SfM 4D) in monitoring ice surface sublimation by comparing it with the weighing pan. The O-T-SfM 4D photogrammetry experiment was conducted in two winter periods of 2023 and 2024. The observations indicate: O-T-SfM 4D monitoring of winter sublimation shows strong agreement with the weighing method. The deviation of ice surface sublimation monitored by O-T-SfM 4D is generally less than 0.09 mm/d (relative error 8%). Owing to its low cost and simplicity, the O-T-SfM 4D photogrammetry method shows strong potential to serve as a reliable tool for monitoring winter ice sublimation.

## Highlights

• SfM photogrammetry method was used to estimate ice sublimation rates by measuring ice depth
• The sublimation results show good consistency between SfM photogrammetry and the weighing pan
• The SfM photogrammetry method can provide spatial-temporal variations of ice sublimation
• The SfM photogrammetry has great potential to be applied in for ice sublimation

## Graphical abstract

**Figure:**
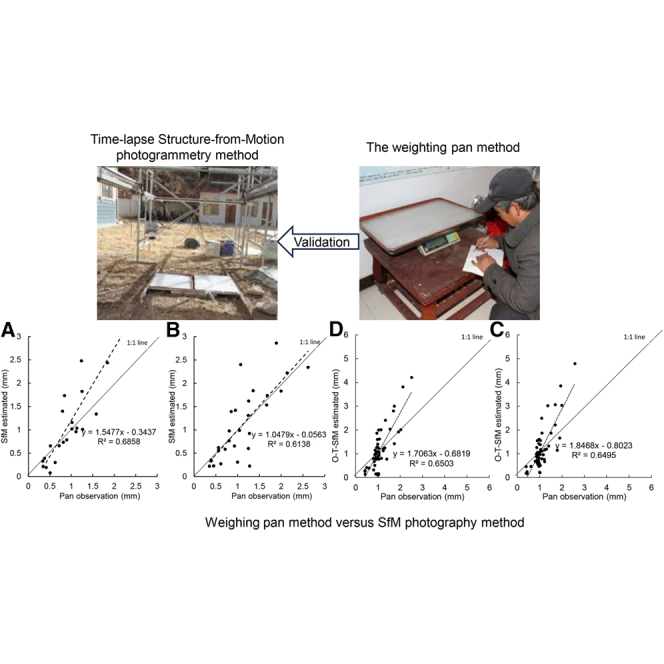

## Introduction

Sublimation is one of the main pathways for the exchange of moisture and energy between snow, glaciers, river ice, lake ice, and sea ice with the atmosphere. Whether it is snow, glaciers, river ice, or lake ice, sublimation plays a significant role and has a profound impact on the mass balance of ice and snow surfaces, energy exchange, and the hydrological processes of cold regions' basins.^1^ Studies have shown that sublimation losses from snow can account for about 10–40% of snowfall.^2^^,^^3^^,^^4^^,^^5^^,^^6^ Sublimation losses from glaciers account for 15–90% of snowfall.^7^^,^^8^^,^^9^ The sublimation of lake ice account to 12.3–23.5% of the total evaporation from lakes.^10^

Sublimation continues to cause mass loss from snow, glaciers, river ice, and lake ice. This exchange of material between the land surface and the atmosphere has important impacts on both the water and energy cycles. However, observational research on snow and ice sublimation is very limited due to the difficulties of continuous observation. The main methods of sublimation observation currently include the weighing method and the eddy covariance method.^11^^,^^12^^,^^13^^,^^14^^,^^15^^,^^16^ Under the premise of no influence from snowfall and blowing snow, the weighing method quantifies sublimation using a sublimation pan or lysimeter by manually weighing the weight change of a snow or ice sample over a period of time. The weighing method is widely used in monitoring sublimation on snow and ice surfaces,^2^^,^^16^ but it is difficult to obtain long sequences of sublimation data in some areas with harsh climate conditions—such as glacier surfaces. Additionally, the sublimation pan used for measurements affect energy exchange between snow, ice, atmosphere and soil, which leads to uncertainties in the weighing observations.^17^

The eddy covariance method calculates latent heat flux by measuring the turbulent pulses of meteorological elements such as wind speed, temperature, and humidity (10∼20 Hz), and then the quantity of sublimation, evaporation, or condensation on the ice surface is derived based on the latent heat coefficient of snow/ice sublimation or evaporation.^11^^,^^13^^,^^14^ This method is recognized as the most reliable observation method, capable of providing high-resolution sublimation observational data. However, the equipment is expensive, and it is susceptible to low temperatures, condensation, and deposition, resulting in poor stability. It often requires additional heating to de-ice the probe to maintain normal operation.^18^ Moreover, the low temperature and smooth characteristics of snow and glacier surfaces result in a lower level of atmospheric turbulent exchange, which brings significant uncertainty to the eddy covariance quantification of sublimation.^19^^,^^20^ Given the harsh winter climate conditions, this extreme environment restricts the continuous observation capabilities of both the weighing and eddy covariance methods, resulting in a severe lack of observational sublimation data.

Simulating snow and ice surface sublimation is an important method to compensate for insufficient sublimation observations, but there is considerable uncertainty in the simulation of ice surface sublimation, which requires long-term observational data to verify models.^9^^,^^21^ There are many input variables for snow and ice sublimation simulation studies. The completeness, continuity, and sample size of the input data can cause significant uncertainty in simulation results.^22^ For example, sublimation simulation requires a variety of meteorological observations, which are often not available. Furthermore, the quantification of key parameters in sublimation simulation, such as glacier roughness, is difficult,^23^^,^^24^ which also introduces uncertainty into sublimation simulations. In addition, there are deficiencies in the modeling of complex processes such as wind-blown snow sublimation. Therefore, comparative research results of different snow and ice surface sublimation models show great variability, and no single model performs excellently in all regions.^25^

Structure from motion (SfM) photogrammetry is a technique that provides quantitative observational data for monitoring land surface processes by observing changes in landform morphology. In snow, river ice, and glacier monitoring, the volumetric method refers to quantifying the accumulation or loss of snow and ice surfaces by measuring volumetric changes in the depth of snow or ice. The SfM photogrammetry volumetric method is widely used for quantifying the accumulation and melting of ice and snow,^26^^,^^27^ with most monitoring accuracies at the centimeter level.^28^ Currently, the SfM photogrammetry volumetric method has been used for monitoring snow surface sublimation,^29^ but it has not been used to quantify sublimation on ice surfaces. Given that ice surfaces have fewer features and higher monitoring difficulties, and the ice surface thickness changes caused by sublimation are often less than 1 mm,^30^^,^^31^^,^^32^^,^^33^^,^^34^ the existing SfM monitoring accuracy is insufficient to meet the precision requirements for ice sublimation monitoring. In this study, excess solar energy absorbed by the edges of the stainless steel pan resulted in rapid sublimation along the pan’s perimeter. The resulting cracks present a significant challenge for precise measurement using the SfM method.

In medical research applications, SfM photogrammetry has been used to obtain sub-millimeter-level 3D models of skulls and teeth.^35^^,^^36^ Similarly, for monitoring micro-scale splash erosion and bare soil, sub-millimeter accuracy has also been achieved.^37^^,^^38^ SfM methods have also realized sub-millimeter accuracy in indoor surface deformation monitoring.^39^ To improve the precision of ice surface SfM photogrammetry, the following measures were implemented in this experiment: (1) a complex image network geometry was applied at a photography distance of 0.6 m to capture the ice surface; (2) photogrammetric surveys were conducted in the morning, when the ice surface was not exposed to direct sunlight; and (3) the camera was equipped with circular polarizing filters to enhance the quality of the ice photographs. Through high-precision ice surface elevation models, the study calculates the daily ice surface sublimation rate. To verify the reliability of SfM photogrammetry for monitoring sublimation rate, this research also employs the weighing method for comparative validation against the results from the SfM photogrammetry method. It provides a detailed analysis of the main factors that cause errors in monitoring sublimation by using SfM photogrammetry. At the same time, the study offers some precautions and necessary operating methods to improve the accuracy of sublimation monitoring with SfM photogrammetry.

## Results

### Surface elevation changes of O-T-SfM 4D observation pans I and II

**Figure 1:**
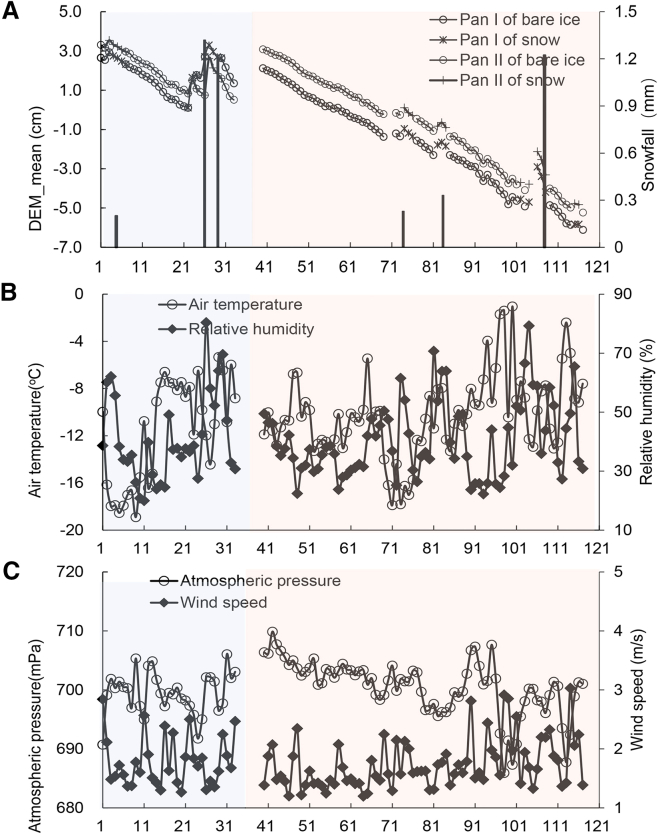
Changes in the average DEMs of Pan I and Pan II obtained by O-T-SfM 4D, together with snowfall, air temperature, relative humidity, atmospheric pressure, and wind speed in two winter periods The first study period from January 12 to February 20, 2023, is a light blue shaded area, and the second study period from December 21, 2023, to March 5, 2024, is a light orange shaded area. (A) Average DEM measurements and snowfall in two winter periods. (B) Daily variations of air temperature and relative humidity in two winter periods. (C) Daily variations of atmospheric pressure and wind speed in two winter periods.

The DEM of the pans over the course of two consecutive winters has shown that the DEM demonstrates a daily decreasing trend on days without snowfall. Dry and sunny weather is conducive to the continuous sublimation of the ice surface, which in turn leads to a consistent reduction in the ice-filled pan DEM (Figure 1). The measurement results from January 12 to February 20, 2023, indicate that the ice thickness of Pan I decreased by 2.0 cm, and the thickness of Pan II decreased by 1.8 cm. During the bare ice period, the rate of ice thickness reduction for Pan I was about 1.25 mm/d, and for Pan II it was about 1.24 mm/d.

After snowfall, with snow covering the ice surface in the pans, the rate of snow thickness reduction for Pan I was about 3.3 mm/d, and for Pan II it was about 3.2 mm/d (Figure 1A). Meteorological observations showed that temperature, relative humidity, atmospheric pressure, and wind speed were −12.0 °C, 41.7%, 699.5 mPa, and 1.8 m/s, respectively, from January 12 to February 20, 2023 (Figures 1B and 1C). Observational results from December 21, 2023, to March 3, 2024, indicated that the thickness of Pan I decreased cumulatively by 8.2 cm, and Pan II decreased by 8.3 cm. Among these, the rates of thickness reduction during the bare ice period for Pans I and II were 1.32 mm/d and 1.33 mm/d, respectively. After snowfall, the snow thinning rates for Pans I and II were 3.4 mm/d and 3.5 mm/d, respectively (Figure 1A). The DEM decrease rate of the snow surface was consistently higher than that of the ice surface. Meteorological observations showed that the temperature and relative humidity were −13.1 °C, 40.3% 700.6 mPa, and 1.7 m/s, respectively, from December 21, 2023, to March 3, 2024 (Figures 1B and 1C).

### Validating O-T-SfM 4D estimated sublimation rate with the weighing method

**Figure 2:**
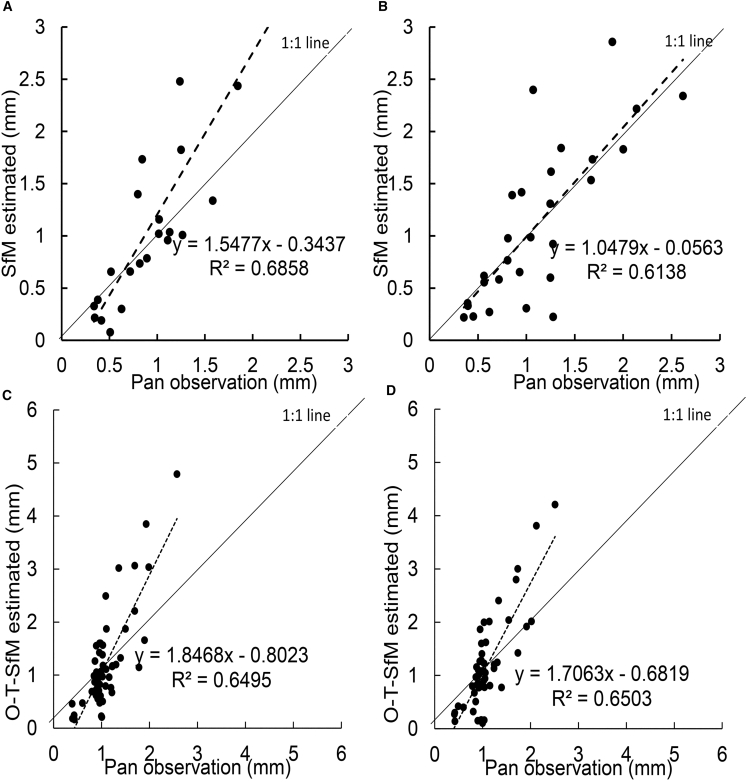
Scatterplot of daily surface sublimation rate from the weighing method versus estimates based on the O-T-SfM 4D method (A and B) are the comparison results for Pan I and Pan II, respectively, from January 12 to February 20, 2023. (C and D) are the comparison results for Pan I and Pan II, respectively, from December 21, 2023, to March 3, 2024.

The analysis shows that the ice surface sublimation rate monitored by the O-T-SfM 4D photogrammetry is in good agreement with the results observed by the weighing pan. The comparative experiment from January 12 to February 20, 2023, indicates that the daily sublimation quantity of Pan I estimated by O-T-SfM 4D has a linear correlation coefficient of 0.83 with the weighing method (Figure 2A). The daily sublimation rate of Pan II estimated by O-T-SfM 4D has a correlation coefficient of 0.78 with the daily sublimation rate acquired by the weighing method (Figure 2B). The results estimated by the O-T-SfM 4D method are significantly linearly correlated with the daily sublimation rate estimated by the weighing method (*p* < 0.01). Using the O-T-SfM 4D method, the estimated average daily sublimation rate for Pan I is 1.08 mm/d compared to 0.97 mm/d estimated by the weighing method, with an average observation error of only 0.06 mm/d for O-T-SfM 4D. The O-T-SfM 4D method estimated the average daily sublimation rate for Pan II as 1.11 mm/d, whereas the weighing method result is 1.11 mm/d, with a measurement error less than 0.01 mm/d for O-T-SfM 4D. Thus, it is evident that the O-T-SfM 4D method shows good consistency with the ice surface sublimation values calculated by the weighing method.

Comparative analysis from December 20, 2023, to March 3, 2024, indicates that the daily sublimation rate from Pans I and II estimated by the O-T-SfM 4D method has a linear correlation coefficient of 0.81 with the weighing method (Figures 2C and 2D). The sublimation rates estimated by O-T-SfM 4D are significantly correlated with the sublimation measurements observed by the weighing method (*p* < 0.01). The sublimation rate for Pan I estimated by O-T-SfM 4D is 1.19 mm/d, compared to the sublimation rate of 1.08 mm/d observed by the weighing method, with an average error of only 0.11 mm/d for the O-T-SfM 4D results. The sublimation rate for Pan II estimated by O-T-SfM 4D is 1.19 mm/d, whereas the weighing method’s observed result is 1.08 mm/d, with an average error of 0.11 mm/d for the O-T-SfM 4D method. The comparative analysis shows that the O-T-SfM 4D estimation results are slightly higher than the weighing method observations. Analysis of sublimation pan photos from December 20, 2023, to March 7 revealed the presence of white ice on the surface, indicating a high concentration of bubbles within the pan ice during this period. When bubble content is high, the ice density typically falls below 0.9 g/cm^3^, causing O-T-SfM 4D estimates to be higher than those obtained from weighing. Based on ice volumes measured by photogrammetry and weights obtained through weighing, the recalculated ice density during these bubble-rich periods was approximately 0.85 g/cm^3^. When this corrected density is applied, the O-T-SfM 4D estimates show excellent agreement with the weighing method.

## Discussion

### Reliability of the O-T-SfM 4D photogrammetry method

The sublimation rates observed by the O-T-SfM 4D photogrammetry over two winters show that O-T-SfM 4D has a higher reliability. The average sublimation rate for the two winters is approximately 1.16 mm/d, while the sublimation rate measured by the weighing pan is about 1.07 mm/d. Using the weighing method as the standard, the error of the O-T-SfM 4D photogrammetry method is 0.09 mm/d, with a relative error of about 8%. This demonstrates that the O-T-SfM 4D photogrammetry method can be used to monitor ice surface sublimation during winter.

Compared with errors in the sublimation monitoring by the weighing method,^40^ Winkler estimated a relative error of 10% for the weighing method,^41^ and Froyland et al. estimated an error of 1 mm/month for the weighing method.^42^ Additionally, related studies indicate that using the eddy covariance method to observe sublimation on snow and ice surfaces has uncertainties of ±10% to ±20%.^18^^,^^43^^,^^44^^,^^45^ Comparing the sublimation error observed by the O-T-SfM 4D photogrammetry method in this study with the results of existing researches by using the weighing method and eddy covariance method, it can be seen that the observation error of the photogrammetry method is 0.09 mm/d with a relative error of 8%, which is superior to the 10% relative error of the weighing method and the ±20% relative error of the eddy covariance method. At present, O-T-SfM 4D photogrammetry for sublimation monitoring has been conducted in relatively stable mountain canyon environments, where the influence of adverse weather conditions such as strong winds and snowfall is minimal. This setting has objectively facilitated the acquisition of high-precision microtopographic data. However, under natural conditions such as fresh snow or clean ice surfaces, the application of O-T-SfM 4D photogrammetry to monitor sublimation still faces significant challenges.

### The uncertainty sources of the O-T-SfM 4D

**Figure 3:**
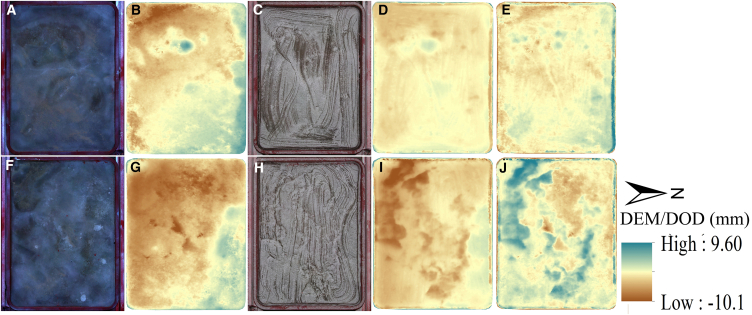
DEMs and corresponding changes of Pan I and Pan II ice surfaces from February 10 to February 21, 2024 (A) Orthoimage of Pan I on February 10. (B) DEM of Pan I on February 10. (C) Orthoimage of Pan I on February 21. (D) DEM of Pan I on February 21. (E) Topographic changes of Pan I caused by sublimation from February 10 to 21. (F) Orthoimage of Pan II on February 10. (G) DEM of Pan II on February 10. (H) Orthoimage of Pan II on February 21. (I) DEM of Pan II on February 21. (J) Topographic changes of Pan II caused by sublimation from February 10 to 21.

In this comparative observation experiment, metal pans were used, which would be a main factor causing errors in the O-T-SfM 4D estimates. Firstly, ice sublimation occurred rapidly, creating cracks on the continuously non-melting ice surface near the interior edges of the pan (Figure 3). These ice cracks at the edges of the pan extended to the bottom, but the O-T-SfM 4D method had difficulty accurately detecting these narrow and deep cracks. This led to an underestimation of the actual sublimation rate by the O-T-SfM 4D photogrammetry, resulting in a lower sublimation rate compared to the weighing method in these cold and non-melting days. For example, Figure 3 shows the cumulative changes in the ice-filled pan DEM from February 10 to 21, 2024. From the DOD visible in Figures 3E and 3J, A high-value sublimation area is clearly formed along the northern edges of the two pans, reaching up to 11 mm. Cracks formed by this sublimation were often narrow and deep, difficult to accurately detect by the O-T-SfM 4D method, causing an underestimation of the actual sublimation in areas with cracks by O-T-SfM 4D in non-melting days. In non-melting days, the air temperatures were low and actual ice sublimation was low, the O-T-SfM 4D method estimated lower sublimation rates than weithting pan (Figure 2).

When melting occurred on the ice surface of the pans, the meltwater flowed into cracks at the edges of the pan, leading to an overestimation of ice surface sublimation by the O-T-SfM 4D method. Although the average temperatures during both observation periods were below 0 °C, half-hourly temperature measurements at the experimental site showed that between January 12 and February 20, 2023, there were 13 days when the highest daily temperature exceeded 0°C. Similarly, from December 21, 2023, to March 3, 2024, there were 28 days when the highest temperature exceeded 0°C. During periods of persistently low temperatures with no melting, cracks formed between the ice and the edges. However, when the temperatures suddenly rose, and the daytime ice surface meltwater flowed into the cracks, this led to rapid decreases in ice surface elevation in the pans, causing the O-T-SfM 4D photogrammetry to monitor a higher sublimation rate than the weighing method. Since the periods when ice surface melting occurred coincided with a higher sublimation rate. Figure 2 shows that the O-T-SfM 4D method tended to overestimate actual ice sublimation when the actual sublimation was high in warm days.

For the operability of the comparative experiment, stainless steel ice-filled pans were used in this test. Cracks caused by rapid sublimation at the edges of the pan created biases in sublimation monitoring by the O-T-SfM 4D method. However, on natural ice surfaces, such cracks would not form due to sublimation, and there would not be the effect of accelerated sublimation at pan edges. It is thus inferred that on natural ice surfaces, the monitoring accuracy of the O-T-SfM 4D photogrammetry method would be unaffected by cracks.

### Limitations of the study

Despite the advantages shown by the O-T-SfM 4D method in estimating ice sublimation, there are also some limitations. Firstly, the observational capability is insufficient for clean ice surfaces. This is mainly due to the lack of effective feature points on such surfaces, leading to insufficient accuracy of the 3D terrain reconstructed by the O-T-SfM 4D method, which affects the continuity of ice surface sublimation in winter. To enhance the feature points on the ice surface while minimizing interference with the natural ice surface remains one of the pending issues for ice surface sublimation photogrammetry. Forward and backward scattering on the ice surface is quite evident, choosing a time without direct sunlight for ice surface photography can improve photo quality and achieve higher photogrammetry accuracy. Additionally, increasing feature points around the surveyed area,^46^ combined with the latest photogrammetry technology,^47^ can improve the accuracy within the surveyed region.

**Figure 4:**
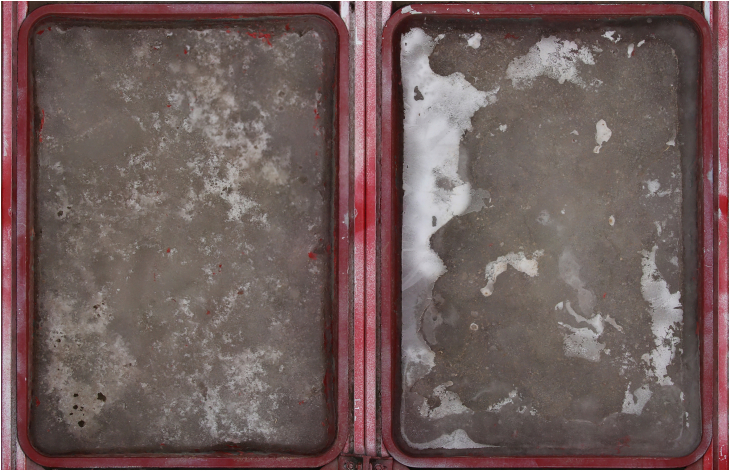
The melting and refreezing of the ice surface within the pan formed a hollow white ice structure, which in turn induced errors into the O-T-SfM 4D sublimation monitoring

Complex structures such as cracks and bubbles in the ice surface can affect the accuracy of sublimation estimation by O-T-SfM 4D photogrammetry (Figure 4). Thus, it is recommended in practice to select ice surfaces that are not affected by bubbles and cracks and use the DOD method to calculate sublimation on the ice surface. By carefully choosing the area of the ice surface being measured, even more accurate O-T-SfM 4D sublimation estimations can be obtained.

One natural ice surface, melt seriously disturbs the accurate estimation of sublimation rates. This is because when snow and ice melt, volume changes include both sublimation and melting information, and the O-T-SfM 4D photogrammetry method cannot differentiate between the two. Hence, direct sublimation rates cannot be obtained with O-T-SfM 4D during warm periods when ice surface melt occurs. In high-altitude and high-latitude regions, during the long winter, snow and ice do not melt. In these areas, the O-T-SfM 4D method can be used to estimate sublimation rates. This method compensates well for the shortcomings of the weighing and eddy covariance methods, which are not sufficiently capable of observations in winter.

At present, sublimation monitoring using photogrammetry is limited to small areas and is only feasible at the plot scale. Because monitoring accuracy typically decreases with increasing imaging distance,^31^ O-T-SfM 4D is not yet suitable for large-area sublimation monitoring. However, with improvements in UAV-based photogrammetry and related techniques, SfM holds promise for achieving sublimation monitoring at much larger spatial scales.

## Resource availability

### Lead contact

Requests for further information and resources should be directed to and will be fulfilled by the lead contact, Rensheng Chen (crs2008@lzb.ac.cn).

### Materials availability

This study did not generate new unique reagents.

### Data and code availability

• All data reported in this article will be shared by the lead contact upon request.
• This article does not report original code.
• All software’s used in this study are commercially available.
• Any additional information required to reanalyze the data reported in this article is available from the lead contact upon request.

## Acknowledgments

We are very thankful to the anonymous reviewers whose comments helped improve the article. This work was supported by the 10.13039/501100001809National Natural Science Foundation of China (42471519, 42271154), the Northwest Institute of Eco-Environment and Resources of 10.13039/501100002367Chinese Academy of Sciences open experimental platform foundation (SJ202414), the 10.13039/501100004775Natural Science Foundation of Gansu Province (24JRRA708), and the “Hundred Talent Program” of the 10.13039/501100002367Chinese Academy of Sciences (Y729G01002).

## Author contributions

Prepared the article: J.L.; help in writing: S.M.; initiated the study: J.L., R.C., and C.H.; carried out field observations: X.W. and Y.L.

## Declaration of interests

The authors declare no competing interests.

## STAR★Methods

### Key resources table

**Table:** 

REAGENT or RESOURCE	SOURCE	IDENTIFIER
**Deposited data**		

Raw and analyzed data	This paper	http://hhsy.casnw.net/

**Software and algorithms**		

Agisoft PhotoScan	Agisoft	https://metashape.cnh
ArcMap	Esri	https://desktop.arcgis.com/zhcn/desktop/

### Experimental model and study participant details

This study does not involve any living participants.

### Method details

#### Study site

The experimental site is located at the standard meteorological field of the Qilian Alpine Ecology and Hydrology Research Station in the upstream area of the Heihe River in the Qilian Mountains (99° 53.1ʹ E, 38° 6.3ʹ N, 2980 m). Two 70 cm×50 cm stainless steel pans were placed on the grassland at the study site.

A 120cm×80 cm aluminum alloy frame was installed around the 2 pans as the control field. Twelve cross-shaped screws on the frame served as ground control points, and their positions were measured with a vernier caliper, achieving an accuracy of 0.02 mm ± 0.02 mm. The control field and the two pans were directly below the mobile platform of the One-Camera Time-Lapse SfM Photogrammetry instrument (O-T-SfM 4D) (Figure S1). The evening prior to the experiment, 15 kg of water was added to each pan and allowed to freeze overnight. By the next morning, the water had solidified into ice, enabling the weighing and O-T-SfM 4D photogrammetry experiments to begin at 9:00. Surface image data of the ice-filled pans were captured using the O-T-SfM 4D measuring device.

Observational comparative experiments were carried out from January 12 to February 20, 2023, and from December 21 2023, to March 5 2024. During the period from January 12 to February 20, 2023, the artificial meteorological field recorded 4 snowfall events with a total snowfall of 5.1 mm, with the maximum snowfall event reaching 3.1 mm. During the experiment, the daily average temperature fluctuated between -5.2 and -24.6 °C, the relative humidity was 41%, and the average wind speed was about 1.8 m/s. From December 21 2023, to March 5 2024, the meteorological field observed 5 snowfall events, with a total snowfall of 6.5 mm and the heaviest snowfall event reaching 3.5 mm. The daily average temperature during the experimental period varied between -2.4 and -23.0 °C. The relative humidity was 40%, and the average wind speed was 1.7 m/s.

#### O-T-SfM 4D photogrammetry

The O-T-SfM 4D photogrammetry instrument used in this experiment is similar to the one used for observing bare ground at the upstream Heihe station^38^ (Figure S1). The camera movement adopted a lawn-mowing pattern, taking a total of 49 photos. Given the sparse snowfall at the monitoring site, the ice surface remained largely unaffected at 0.6 m, and the photography height was maintained at approximately 0.6 m above the ice surface. A more complex image network geometry was used in this experiment (Figure S2): 24 peripheral photos were taken with an inclined angel focus towards the centre of the control field, and the rest of 25 photos were taken with a vertical downward angle. The selected camera for this photogrammetry was a Canon 6D, equipped with a Canon EF 18-108 mm lens fixed at 18 mm. The camera’s shutter speed was set to 1/320 s, and the photo resolution was 3648×5472 pixels. The time of photographying was set at 9:00 AM Beijing time. The O-T-SfM 4D photogrammetry experiment was conducted in two periods: from January 12 to February 20, 2023, and from December 21, 2023, to March 5, 2024, covering a total of 109 days of ice surface imaging.

This study used Agisoft PhotoScan software for photogrammetry data processing. Since the processing mode in PhotoScan can also affect measurement accuracy,^35^ the ultra high processing method in PhotoScan was uniformly applied to all frames, with other settings following the default configuration of PhotoScan. Based on the photos acquired by O-T-SfM 4D, we first generated a dense point cloud using PhotoScan software. We then created the Digital Elevation Model (DEM) data from the dense cloud. Using the boundaries of Pan I and Pan II, we clipped the DEM within each pan using ArcMap software. Subsequently, we calculated the mean DEM and snow depth difference between DEM (DOD) values for each pan using ArcMap. A computer with 64 GB memory and a 3.4 GHz processor was used for processing and analyzing the frames.

#### Sublimation rate measurement with weighing of ice-filled pans

To validate the precision of sublimation rate estimated by O-T-SfM 4D photogrammetry, this study first conducted comparative experiments: at 9:00 AM each day, after the O-T-SfM 4D had completed its photography, the two ice-holding pans were immediately weighed to obtain their daily weight. The daily sublimation rate can be determined by subtracting the weight of the following day from the weight of the previous day. The sublimation rates obtained by the weighing method serve as a standard to verify the reliability of the sublimation rates estimated by O-T-SfM 4D photogrammetry.

### Quantification and statistical analysis

#### Daily surface sublimation rate estimation with O-T-SfM 4D

The estimation of daily snow surface sublimation rate is based on the difference between the DEMs for two sequential days observed by SfM (Equation 1) and the ice density (Equation 2):

(Equation 1) $${DOD}_{t+1}\left(i,j\right)={DEM}_{t+1}\left(i,j\right)-{DEM}_{t}\left(i,j\right)$$

(Equation 2) $$S_{t+1}=\frac{1}{n\times m}\sum_{i=1}^{n}\sum_{j=1}^{m}\left({-DOD}_{t+1}\left(i,j\right)\right)\times \rho_{t+1}\left(i,j\right))$$

where DOD _*t*+1_ (*i*, *j*) is the snow depth difference between DEM(*i*, *j*) at *t* + 1 day and DEM _*t*_ (*i*, *j*) at day *t* (cm); *i* is the *i* th row of the selected DEM and DOD plot (*i* ≤ *n*); and *j* is the *j* th column of the DEM and DOD plot (*j* ≤ *m*). *S*_*t+*1_ is the mean of the estimated daily surface sublimation rate at *t* + 1 day in an *n* × *m* sized plot (mm/day); and ρ_*t+*1_ (*i*, *j*) is the ice or snow density at *t* + 1 day (g/cm^3^). During the study period, ice density was set to 0.9 g/cm^3^. Snow density was measured with gravimetric method in the field with high-precision scale (with a stated accuracy of ±0.1 g) after snowfall.

The thermal deformation of the aluminum alloy frame applied in this study as control frame, could potentially influence O-T-SfM 4D photogrammetry accurcy. The daily snow surface sublimation rate was corrected based on the ground surface temperature observed at the photogrammetric measurement time and the coefficient of linear thermal expansion for aluminum (α = 23 × 10^-6^ /°C).

#### Data statistics and analysis

Air temperature, Relative humidity, Atmospheric pressure and wind speed were directly observed from automatic weather station at the experimental site. Correlation analyses among Pan observed sublimation rates and O-T-SfM 4D estimated sublimation rates were performed through linear correlation methods.
