# Supplementary material for: Investigating the capability of the structure-from-motion photogrammetry on monitoring the ice surface sublimation
Source: iScience. 2026 Jan 24;29(2):114775. doi: 10.1016/j.isci.2026.114775 (PMC12919286; doi:10.1016/j.isci.2026.114775)
Supplement: Document S1. Figures S1 and S2 [file mmc1.pdf]

iScience, Volume *29*

Supplemental information

Investigating the capability of the structure-from-motion photogrammetry on monitoring the ice surface sublimation

Junfeng Liu, Shaoxiu Ma, Rensheng Chen, Yongyuan Li, Xueliang Wang, and Chuntan Han

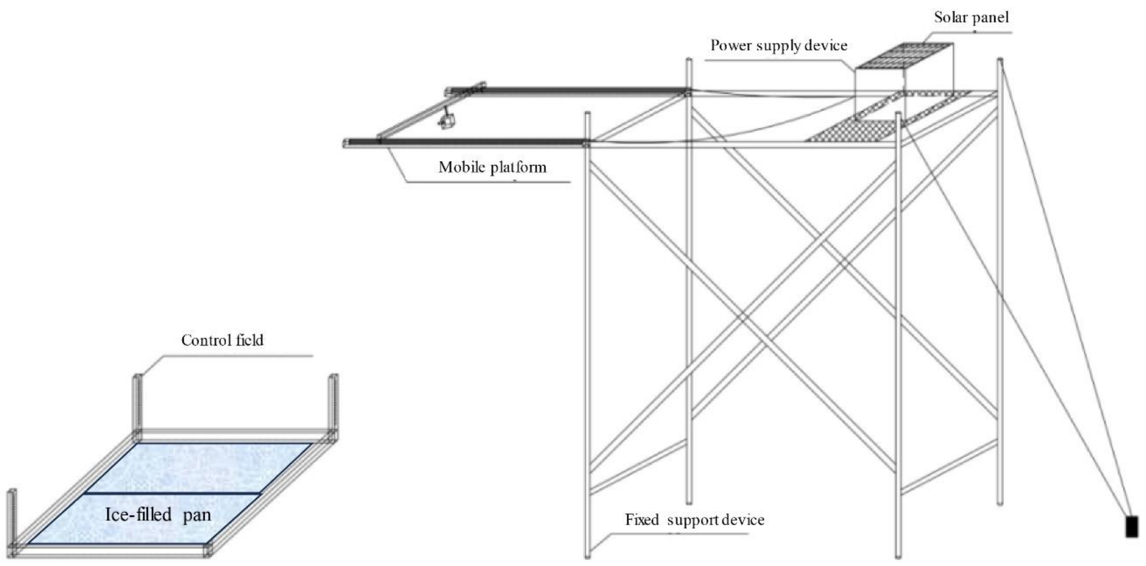

Figure S1. Comparison experiment of ice-filled pan measured by using O-T-SfM 4D photogrammetry and weighing pan

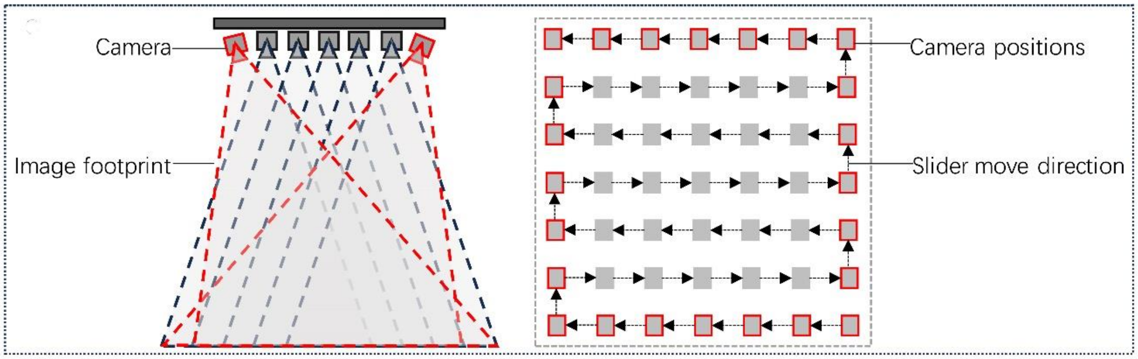

Figure S2. Schematic diagram showing the O-T-SfM 4D setup used to collect experimental photos for the ice-filled pans experiments, in which read rectangular of peripheral photos were taken with an inclined angel focus towards the centre of the control field and ice-filled pans, and the gray rectangular of central 25 photos were taken with a downward angle
